# Identification of common genes and biomarkers between Dermatomyositis and rheumatoid arthritis through integrated bioinformatics

**DOI:** 10.1371/journal.pone.0340617

**Published:** 2026-02-04

**Authors:** Fo Yang, Qishui Xia, Mingjun Wu, Haibo Hu, Jiuchu Jin, Tianpeng Chen, Wei Fan, Gongtao Jiang

**Affiliations:** Nanchang Hongdu Hospital of Traditional Chinese Medicine, Nanchang, China; Independent Medical Researcher and Writer, UNITED KINGDOM OF GREAT BRITAIN AND NORTHERN IRELAND

## Abstract

**Background:**

Dermatomyositis (DM) and rheumatoid arthritis (RA) share immuno-inflammatory features, yet mechanisms underlying their comorbidity remain unclear. We aimed to define shared molecular mechanisms across gene regulatory networks and the immune microenvironment using integrated multi-omics and machine-learning analyses.

**Methods:**

Microarray datasets for RA (GSE55235, GSE55457, GSE12021) and DM were retrieved from GEO. RA datasets were merged and batch-corrected with ComBat. Differentially expressed genes (DEGs) were identified using limma; key modules were derived by weighted gene co-expression network analysis (WGCNA). Intersected DEGs–module genes underwent GO/KEGG enrichment. Core genes were prioritised by LASSO regression and random-forest modelling and evaluated in external cohorts. Immune landscape was estimated with CIBERSORT and immune subpopulations profiled by single-sample GSEA. Single-cell RNA-seq (GSE159117) mapped cell-type–specific expression of core genes and inferred ligand–receptor networks.

**Results:**

We identified 780 DEGs in RA and 739 in DM. Intersecting DEGs with WGCNA modules yielded 47 candidates enriched for IL-17, Toll-like receptor and chemokine signalling (all *P* < 0.05). Four core genes (JUNB, NRGN, HCP5, RARRES3) were prioritised; HCP5 and RARRES3 showed significant differential expression and diagnostic performance in external datasets (AUC 0.634–0.846). CIBERSORT indicated enrichment of activated CD4^+^ memory T cells and a shift in macrophage polarisation with increased M2 signatures in both diseases. Core genes were dynamically associated with M1/M2 polarisation and T-cell subpopulations (*P* < 0.05). Single-cell analysis localised core gene expression to NK cells, monocytes and T/B cells, and highlighted inflammatory ligand–receptor interactions.

**Conclusions:**

Integrative, ML-assisted transcriptomics reveals convergent RA–DM programmes centred on IL-17, TLR and chemokine pathways with remodelling of the immune microenvironment. HCP5 and RARRES3 emerge as reproducible, externally supported candidates with diagnostic potential and plausible links to macrophage polarisation and T-cell states. These findings nominate testable biomarkers and pathways for validation and provide a rationale for pathway-guided, cross-disease studies of RA–DM comorbidity.

## Introduction

Dermatomyositis (DM) is a prototypical idiopathic inflammatory myopathy in which immune-mediated myopathy coexists with characteristic cutaneous stigmata, and in a proportion of patients there is lung, cardiac or other organ involvement [1]. Rheumatoid arthritis (RA) is a chronic, systemic autoimmune disease defined by persistent synovitis that damages cartilage and bone and is frequently complicated by extra-articular manifestations, including pulmonary disease. These complementary clinicopathological definitions motivate a comparative interrogation of shared mechanisms that may transcend tissue tropism [2,3].

Despite divergent clinical signatures, converging molecular and cellular evidence points to disease-agnostic axes of pathobiology. Interferon-driven immunity is a defining feature of DM across blood, muscle and skin, with an interferon gene signature also detectable in subsets of RA; in both settings these signals funnel through JAK–STAT transcriptional programmes and correlate with inflammatory activity [4–6]. Vascular injury adds a second layer of commonality: DM muscle exhibits complement-mediated microangiopathy with perifascicular capillary loss and deposition of the terminal complement complex (C5b-9) on endomysial microvessels, although broader neuropathology cautions that C5b-9 deposition is not wholly specific to DM [7,8]. At the level of genetic architecture, immune-regulatory loci are enriched across both diseases—RA risk centred on HLA-DRB1 “shared epitope” alleles and multiple non-HLA variants, and idiopathic inflammatory myopathies showing autoantibody-stratified HLA class II associations—with cross-disease analyses implicating JAK–STAT-adjacent nodes such as TYK2 that are relevant to DM [9–12]. Tissue circuits further reinforce this overlap: pathogenic fibroblast subsets and disease-state macrophage populations in RA synovium orchestrate chronic inflammation, while DM tissues are enriched for plasmacytoid dendritic cells that amplify type I interferon signalling, highlighting shared stromal–immune crosstalk and antigen-presentation pathways [4,13,14]. Clinically, these mechanistic intersections manifest in overlapping complications. Interstitial lung disease is a leading extra-musculoskeletal manifestation of both disorders; anti-MDA5-positive DM can present with rapidly progressive ILD and inflammatory arthritis that mimics RA, and RA-associated ILD remains a major driver of morbidity and mortality [15–19]. Therapeutically, the same axes map to druggable hubs: JAK inhibition is established in RA management and early evidence supports JAK-targeted strategies in DM, including anti-MDA5 DM-ILD, warranting rigorous cross-disease translational analysis [20–22].

In summary, DM and RA exhibit significant overlap at transcriptional, histopathological and clinical levels, suggesting partially shared core molecular pathways. However, most existing studies interrogate each disease in isolation, limiting our ability to define common therapeutic liabilities. This study therefore applies an integrated bioinformatics framework to identify shared differentially expressed genes (DEGs), co-expression modules and upstream regulators across DM and RA; to reconstruct conserved protein–protein and regulatory networks; to deconvolute disease-relevant cell states across blood, synovium, muscle and skin; and to prioritise candidate therapeutics through network- and signature-based repurposing analyses. By delineating the shared molecular architecture of DM and RA, we aim to provide a mechanistic substrate for broad-spectrum therapeutic strategies and for the rational optimisation of existing drug combinations.

## Materials and methods

### Study design

**Fig 1:**
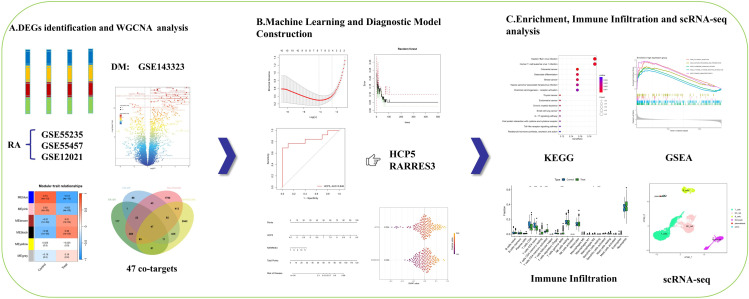
Workflow of the analysis.

This study was a bioinformatics-based investigation designed to explore the shared molecular mechanisms between DM and RA. Publicly available microarray and single-cell RNA sequencing datasets were obtained from the Gene Expression Omnibus (GEO) database for integrative multi-omics analysis. The overall workflow, summarised in Fig 1, comprised the following major analytical steps: data collection and preprocessing, removal of batch effects, identification of DEGs, construction of weighted gene co-expression networks (WGCNA), and determination of intersecting genes between DM and RA. Subsequently, functional enrichment analysis, machine learning–based feature selection, and validation in external datasets were conducted. Immune landscape analysis and single-cell transcriptomic profiling were further integrated to delineate the immune landscape and cell–cell communication patterns.

### Data sources

We queried the NCBI GEO database using keyword combinations (“rheumatoid arthritis”, “dermatomyositis” and “Homo sapiens”) together with tissue filters such as “synovium”, “skeletal muscle”, “whole blood” or “PBMC”, and data type filters “expression profiling by array” and “single-cell RNA-seq”. The retrieved records and their associated publications were screened to exclude non-expression series, non-human studies, datasets without an appropriate control group, and series based on non-target tissues (for example skin for dermatomyositis when focusing on muscle, or in vitro stimulated cells). We also required a clear case–control design in disease-relevant tissue, use of widely adopted microarray platforms with available series-matrix or raw data, and a minimum sample size of approximately 8–10 samples per group for discovery analyses.

Based on these criteria, we shortlisted accession numbers and finally included seven datasets: GSE93272, GSE55235, GSE55457, GSE12021, GSE143323, GSE1551 and GSE159117. For discovery cohorts, we used RA synovial-tissue microarrays (GSE55235: 10 RA and 10 controls; GSE55457: 13 RA and 10 controls; GSE12021: 12 RA and 9 controls) and a DM skeletal-muscle microarray (GSE143323: 39 DM and 20 controls). For independent validation, we used a whole-blood microarray in RA (GSE93272: 232 RA and 43 controls) and a skeletal-muscle microarray in DM (GSE1551: 13 DM and 10 controls). In addition, a single-cell RNA-seq dataset from RA PBMCs (GSE159117) was included for downstream scRNA-seq analyses and cell–cell communication, but not for differential-gene discovery. Case–control status, tissue source and platform information followed the original GEO annotations. Diagnostic ascertainment adhered to the criteria stated by the contributing studies, which align with widely used standards (the 2010 American College of Rheumatology/European League Against Rheumatism classification criteria for RA and the Bohan and Peter criteria for DM), where reported. A consolidated summary of platforms, sample sizes, organisms and tissues, as well as the discovery or validation designation used in our pipeline, is provided in Table 1.

**Table 1:** Detailed information on datasets used in the study.

DataSets	Platforms	Sample Size		Organism	Tissue	Attribute	Population description
		Control	RA/DM				
GSE93272	GPL570	43	232	Homo sapiens	Whole blood	Validation	RA patients and healthy controls
GSE55235	GPL96	10	10	Homo sapiens	Synovial tissue isolated from joint	Training	RA patients and healthy controls
GSE55457	GPL96	10	13	Homo sapiens	Synovial tissue	Training	RA patients and healthy controls
GSE12021	GPL96	9	12	Homo sapiens	Synovial tissue	Training	RA patients and healthy controls
GSE143323	GPL21290	20	39	Homo sapiens	Skeletal muscle	Training	DM patients and healthy controls
GSE1551	GPL96	10	13	Homo sapiens	Skeletal muscle	Validation	DM patients and healthy controls

### Removal of batch effect

We merged three RA datasets (GSE55235, GSE55457, and GSE12021) and applied the “ComBat” function from the “sva” package to correct for batch effects [23]. To assess the impact of this correction, we conducted a comparison of data quality both prior to and following the removal of batch effects, utilizing principal component analysis (PCA) [24].

### Identification of DEGs

Differential expression analysis was conducted using the limma R package, applying its classical microarray linear-modelling pipeline (log_2_ transformation, quantile normalisation, lmFit and eBayes) [25]. DEG analysis was performed separately for each disease. For RA, limma was applied to the batch-corrected, merged synovial datasets (GSE55235, GSE55457 and GSE12021) to compare RA synovium (n = 35) with synovial controls (n = 29). For DM, limma was applied to the skeletal-muscle dataset GSE143323 to compare DM muscle (n = 39) with control muscle (n = 20). Genes with adjusted *P* < 0.05 and |log₂ fold change| > 1.0 in each contrast were defined as DEGs [26]. We then intersected the RA and DM DEG sets to obtain genes that were differentially expressed in both diseases relative to their own tissue-matched controls [27].

### Weighted gene co-expression network analysis

WGCNA analysis was performed on the GSE55235 and GSE143323 datasets using the “WGCNA” package [28]. First, the top 5000 genes with the highest variance were selected, Pearson correlation coefficients were calculated, and an adjacency matrix was constructed. This matrix was then transformed into a Topological Overlap Matrix (TOM). The dynamic tree cut method was applied to identify gene modules with similar expression patterns, with the minimum module size set to 30 genes. Key module genes with the highest correlation coefficients were selected for subsequent analysis.

### Pathway enrichment analysis

The biological functions and signalling pathways of the intersecting genes were interrogated in R using clusterProfiler for over-representation analysis [29]. Gene Ontology (GO) enrichment analysis was performed separately for the three GO domains, including biological process (BP), molecular function (MF) and cellular component (CC). These domains were chosen because they provide complementary information on the roles of differentially expressed genes in DM and RA: BP summarises the immune and inflammatory pathways and other biological processes that are jointly dysregulated, MF characterises the specific biochemical activities of the encoded proteins, and CC indicates their subcellular and extracellular localisation, helping to identify secreted or membrane-associated proteins with biomarker or therapeutic potential. Together, these categories offer an integrated view of how shared genes contribute to common pathogenic mechanisms in both diseases [30]. We used KEGG because its manually curated pathway maps encode metabolic and signalling circuitry with pathway topology, facilitating mechanistic interpretation and network-level visualisation; these resources align with recommended best practices for pathway enrichment and result interpretation. Enrichment GO BP/CC/MF and KEGG was performed with multiple-testing correction, and terms/pathways with Benjamini–Hochberg FDR < 0.05 were considered significant.

GSEA is a powerful statistical method used to identify potential biological patterns in gene expression data. Unlike traditional differential expression analysis, GSEA does not focus on the expression changes of individual genes. Instead, it evaluates the overall performance of predefined gene sets across the samples to determine whether they are significantly enriched between the experimental and control groups. This method calculates the Enrichment Score (ES) for each gene set and controls the significance level through multiple hypothesis testing, thereby identifying gene sets associated with specific biological processes or diseases.

### Machine learning algorithms

We employed two machine learning algorithms to systematically identify the core genes associated with RA and DM from the intersecting genes. LASSO regression, a variant of linear regression, performs variable selection and controls model complexity by applying an L1 regularization term to the regression coefficients. LASSO effectively identifies the most predictive genes while avoiding overfitting, and its inherent sparsity ensures that the selected genes possess high interpretability. Random Forest (RF) is an ensemble learning technique that leverages decision trees to enhance classification accuracy and mitigate overfitting, achieved by constructing a multitude of decision trees and aggregating their outcomes through voting [31]. RF demonstrates strong noise resistance and superior classification performance when handling gene selection problems with many features and complex data structures, enabling the identification of potential key genes. The core genes identified by both machine learning algorithms were intersected, and the results were validated using external datasets GSE93272 for RA and GSE1551 for DM.

### DM and RA risk prediction by core overlapping genes

Using the R package “rms”, we constructed nomogram models for both RA and DM, incorporating the selected core genes as predictive features. Nomograms were chosen because they provide an intuitive and quantitative tool for individualised risk prediction, translating complex regression outputs into a clinically interpretable graphical format that can be readily applied to patient-level decision-making. These models were developed through regression analysis to generate risk-prediction nomograms, and their predictive performance was evaluated using calibration curves, which assess the agreement between predicted probabilities and actual observed outcomes [32,33].

To interpret the machine learning model, we first trained a supervised classifier using the log₂-transformed, normalised expression values of the core overlapping genes as input features and the sample-level disease status (RA or DM versus control) as the binary outcome. We then applied Shapley Additive Explanations (SHAP) to the fitted model to quantify how the expression of each gene contributed to the predicted probability of disease for each sample. In this context, positive SHAP values indicate that the observed expression of a gene pushes the prediction towards the RA/DM class, whereas negative SHAP values shift the prediction towards the control class. By summarising SHAP values across all samples, we obtained a quantitative and directionally explicit measure of the relative importance of HCP5 and RARRES3 in driving the model’s predictions [34].

### Associations between core genes and immune cell lineages

Immune cell composition was estimated with CIBERSORT using the LM22 signature (22 leukocyte subsets). Analyses were restricted to samples with CIBERSORT deconvolution *P* < 0.05 [35]. For each core overlapping gene, we quantified its association with each immune-cell fraction using Spearman’s rank correlation within each dataset. Correlation coefficients were Fisher z–transformed and, where multiple datasets were available, pooled by random-effects meta-analysis and back-transformed to r. Multiple testing across all gene–cell pairs was controlled by Benjamini–Hochberg FDR, with FDR < 0.05 considered significant. Because CIBERSORT returns relative fractions (compositional data), we performed a sensitivity analysis using centred log-ratio (clr)–transformed fractions; conclusions were unchanged.

Due to the absence of a DM single-cell dataset, we used the RA dataset GSE159117 for single-cell analysis. This dataset includes scRNA-seq data from peripheral blood mononuclear cells (PBMCs) of a RA patient. Data analysis was performed using the Seurat package in R (version 4.4.1) [36]. First, quality control was conducted on the raw data: cells with fewer than 200 or more than 10,000 detected genes were removed, cells with fewer than 300 or more than 15,000 total RNA molecules were excluded, and cells with mitochondrial gene proportions ≤ 15% were retained. After screening, the top 2,000 genes with the highest expression variance were selected for subsequent analysis. Following data normalization, PCA was performed for dimensionality reduction, and UMAP was used to visualize the cell distribution. Using the FindClusters function in Seurat (resolution = 1), we divided the cells into 16 subgroups and annotated cell types using the singleR package and reference datasets [37]. To further explore cell-cell interactions, we constructed a cell communication network using the CellChat tool [38]. This tool quantifies the signaling pathway strength between different cell subgroups based on known ligand-receptor interaction databases, systematically analyzing the characteristic changes in cell communication within the RA microenvironment.

## Results

### Participant characteristics across included datasets

Before presenting the bioinformatics findings, we summarised the socio-demographic features of participants in the included datasets. Across synovial tissue (RA and controls), skeletal muscle (DM and controls), and whole-blood cohorts, sex and age were inconsistently recorded in the GEO annotations and, where unavailable, are reported as “not reported” in Table 1. Per-dataset sample numbers and tissues are listed to aid interpretability.

### Identification of DEGs associated with RA and DM

**Fig 2:**
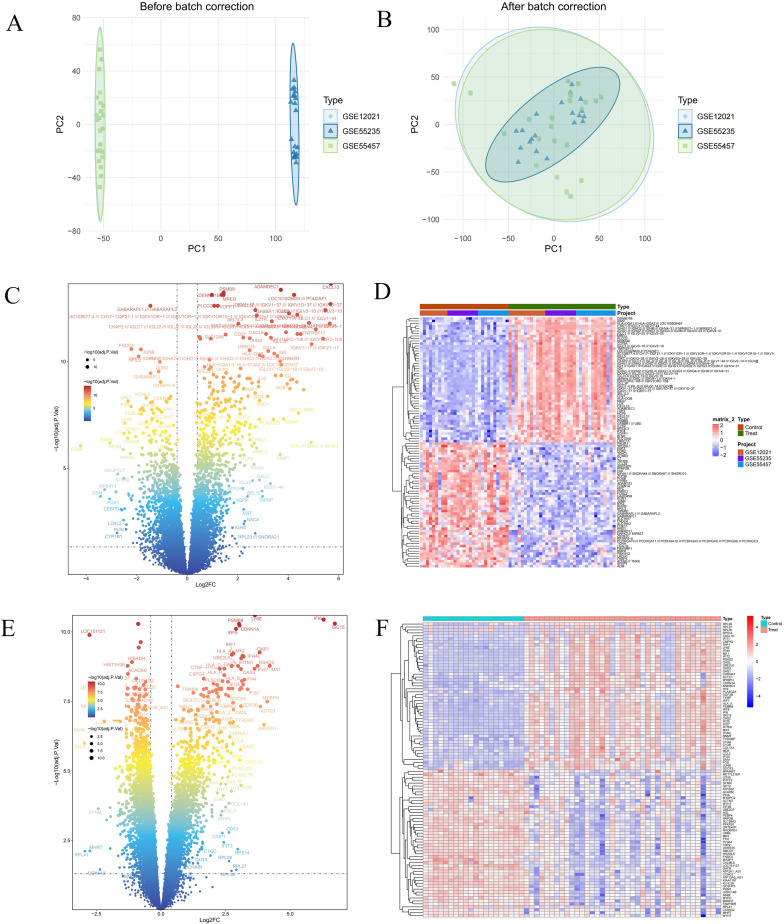
Integration of RA datasets and identified the DEGs. **(A, B)** Two-dimensional PCA cluster plot of GSE12021, GSE55235 and GSE55457 datasets before and after normalization. **(C)** Volcano plot of DEGs. **(D)** Heatmap of DEGs.

The design flowchart is shown in Fig 1. After removing batch effects from the GSE55235, GSE55457, and GSE12021 datasets, they were integrated and normalized, resulting in 35 RA cases and 29 control group samples (Fig 2A, 2B). We performed differential expression analysis using the “limma” package on the combined RA dataset and the DM dataset GSE143323 to identify common pathogenic genes associated with both RA and DM. In the RA dataset, 780 DEGs were identified, with 430 upregulated and 350 downregulated (Fig 2C, 2E). In the DM dataset, 739 DEGs were found, with 485 upregulated and 254 downregulated (Fig 2D, 2F).

### WGCNA analysis identifies key module genes associated with RA and DM

**Fig 3:**
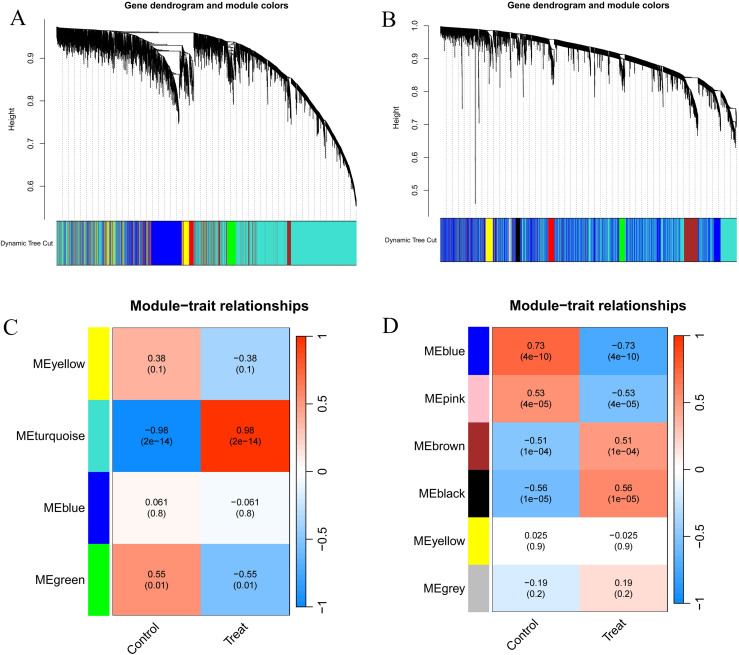
Screening of genes by WGCNA algorithm. **(A, B)** The correlation coefficients between gene modules and ankylosing spondylitis. **(C, D)** Dendrogram of the gene modules. WGCNA, weighted gene coexpression network analysis.

We performed WGCNA on the RA and DM datasets separately. In GSE55235, based on scale independence and average connectivity, we selected a soft threshold of β = 3. Genes were clustered into four modules, with the ‘MEturquoise’ module showing the highest correlation with RA (MEturquoise: r = 0.98, p = 2e − 14) (Fig 3A, 3C). Similarly, in the DM dataset GSE143323, we selected a soft threshold of β = 5, resulting in six modules, with the most significant being the blue module (MEblue: r = 0.73, p = 4e − 10) (Fig 3B, 3D) (S1 File). We therefore defined the MEturquoise module in RA and the blue module in DM as the key disease-related modules and used them for overlap with DEGs and all subsequent enrichment and prioritisation analyses.

### Functional enrichment of common pathogenic genes in RA and DM

**Fig 4:**
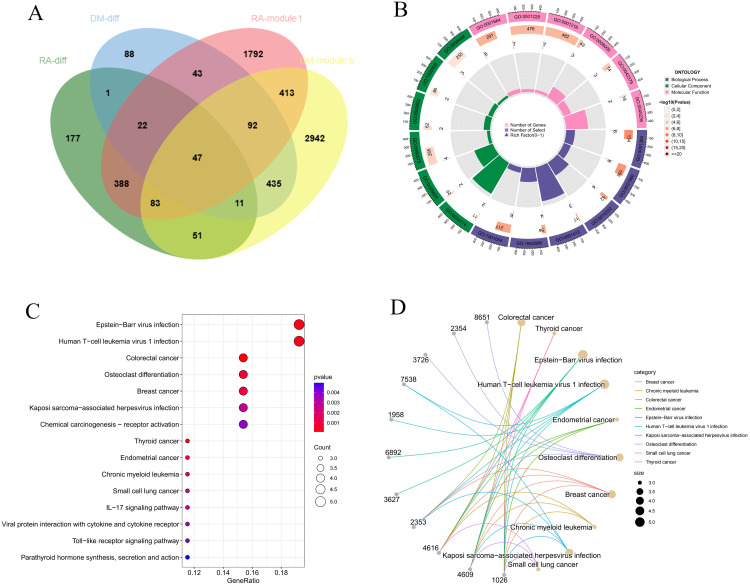
Function enrichment analysis of the intersecting genes. **(A)** The intersecting of DEGs and WGCNA module genes includes 47 genes. **(B)** Circle plot of GO enrichment analysis includes biological process, cellular component and molecular function. **(C, D)** Bubble plot and circle plot of KEGG enrichment.

We intersected the RA DEGs with genes in the RA key module (MEturquoise) and the DM DEGs with genes in the DM key module (blue), and then merged these two sets, resulting in 47 shared candidate genes (Fig 4A). Enrichment analysis was performed using GO and KEGG to identify potential mechanisms of action. GO enrichment analysis revealed that genes highly represented in the response to glucocorticoids, corticosteroids, and corticosterone were enriched. Key cellular components included the RNA polymerase II transcription regulator complex, proteasome core complex, beta-subunit complex, and proteasome core complex. A high proportion of molecular functions were associated with DNA-binding transcription activator activity, RNA polymerase II-specific DNA-binding transcription activator activity, and G protein-coupled receptor binding (Fig 4B). KEGG pathway analysis further revealed that the genes were primarily enriched in signaling pathways such as osteoclast differentiation, IL-17 signaling pathway, and Toll-like receptor signaling pathway (Fig 4C, 4D).

#### Machine learning algorithms were used to select core genes.

**Fig 5:**
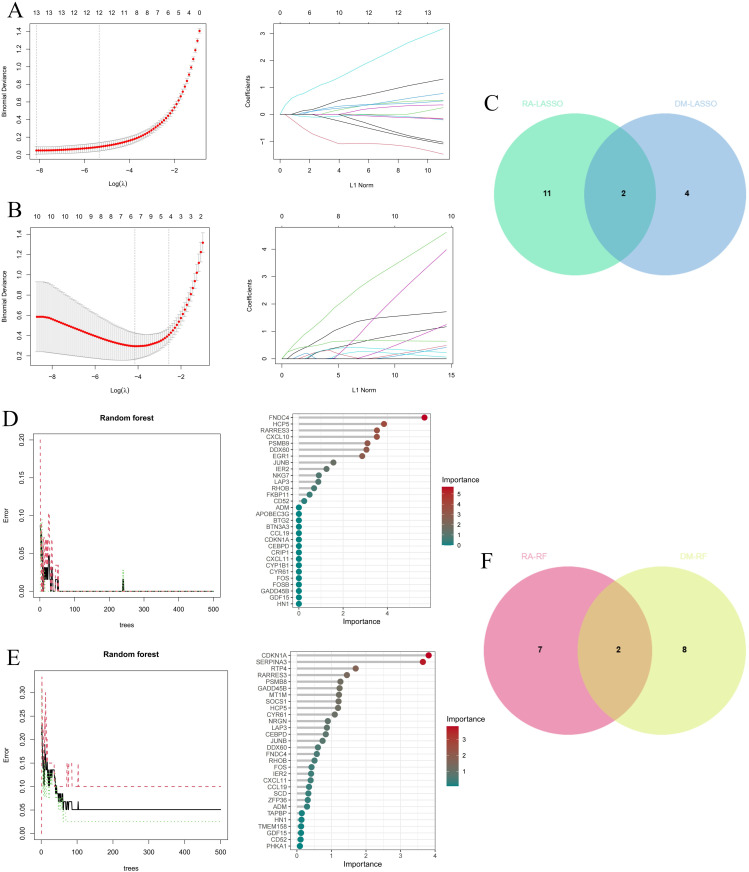
Identification of diagnostic genes using machine learning algorithm. **(A-C)** Feature genes selection through LASSO. **(D-F)** Feature genes selection through RF.

The selection process employed LASSO regression and random forest algorithms. In the LASSO regression analysis, 13 genes were identified from the RA dataset and 6 genes from the DM dataset. After cross-referencing, 2 core genes (JUNB and NRGN) were identified (Fig 5A–5C). Using the random forest algorithm, 9 genes were selected from the RA dataset and 10 genes from the DM dataset. After removing the intersection, the core genes HCP5 and RARRES3 were identified (Fig 5D–5F). By combining the core genes from both algorithms, a final list of 4 core genes was obtained: JUNB, NRGN, HCP5, and RARRES3.

#### Validation of core genes and construction of diagnostic models.

**Fig 6:**
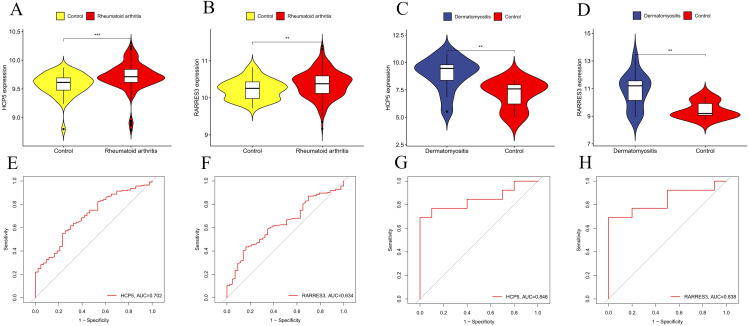
Validation of the expression level and diagnostic efficacy of HCP5 and RARRES3. **(A)** The violin plots of HCP5 in GSE93272. **(B)** The violin plots of RARRES3 in GSE93272. **(C)** The violin plots of HCP5 in GSE1551. **(D)** The violin plots of RARRES3 in GSE1551. **(E)** The ROC curves of HCP5 in GSE93272 **(F)** The ROC curves of RARRES3 in GSE93272 **(G)** The ROC curves of HCP5 in GSE1551. **(H)** The ROC curves of RARRES3 in GSE1551. *p < 0.05; **p < 0.01;***p < 0.001; ****p < 0.0001.

**Fig 7:**
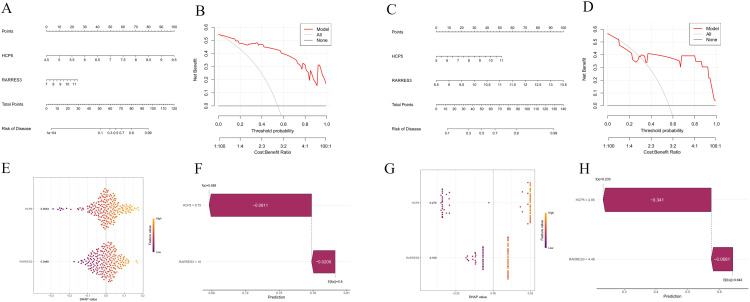
Construction of disease risk models. **(A, B)** Nomogram developed for clinical use, enabling precise prediction of RA risk based on the expression of hub genes. **(C, D)** Nomogram developed for clinical use, enabling precise prediction of DM risk based on the expression of hub genes. **(E, F)** SHAP summary plot of RA model. **(G, H)** SHAP summary plot of DM model.

To further validate the reliability of the core genes, we used GSE93272 and GSE1551 as external datasets. Compared to the control group, the expression of HCP5 and RARRES3 showed significant differences in the RA and DM validation datasets (*P* < 0.05) (Fig 6A–6D). However, the differential expression of JUNB and NRGN was not significant in the validation datasets (S2 File). Additionally, ROC analysis showed that HCP5 provided moderate discriminatory ability in GSE93272 (AUC = 0.704) and good diagnostic performance in GSE1551 (AUC = 0.846) (Fig 6E, 6G). RARRES3 also showed moderate discrimination in GSE93272 (AUC = 0.634) and good diagnostic performance in GSE1551 (AUC = 0.838) (Fig 6F, 6H). To enhance clinical applicability, we developed a predictive model (Fig 7A–7D) that integrates the expression of two core genes to accurately estimate the likelihood of RA and DM. Additionally, we used SHAP values to quantify how the expression of HCP5 and RARRES3 contributed to the predictions of the classification model. The waterfall plots show that samples with the disease-typical expression pattern of these genes exert positive SHAP contributions, shifting the predicted probability towards the RA/DM class (Fig 7E–7H).

### GSEA analysis of core genes

**Fig 8:**
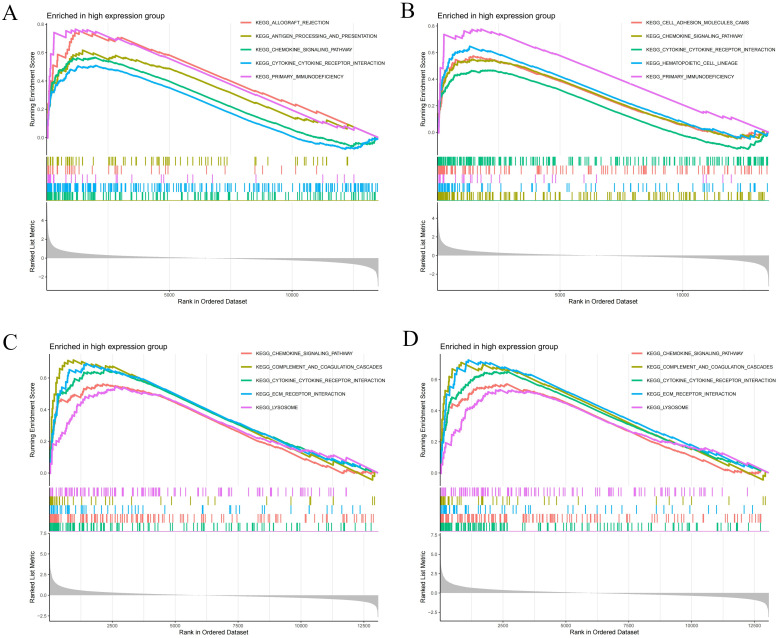
GSEA analyses results. **(A, B)** GSEA analysis results of HCP5 and RARRES3 in RA samples. **(B)** GSEA analysis results of HCP5 and RARRES3 in DM samples.

The GSEA revealed positive enrichment of several pathways in the RA samples, including the chemokine signaling pathway, cytokine-cytokine receptor interaction, and primary immunodeficiency (Fig 8A, 8C). Conversely, the DM samples exhibited positive enrichment in lysosome activity, chemokine signaling, cytokine-cytokine receptor interaction, complement and coagulation cascades, as well as extracellular matrix receptor interaction (Fig 8B, 8D). Notably, both RA and DM shared common pathways, namely the cytokine-cytokine receptor interaction and chemokine signaling pathway, which were enriched in both conditions.

### Immune landscape analysis results

**Fig 9:**
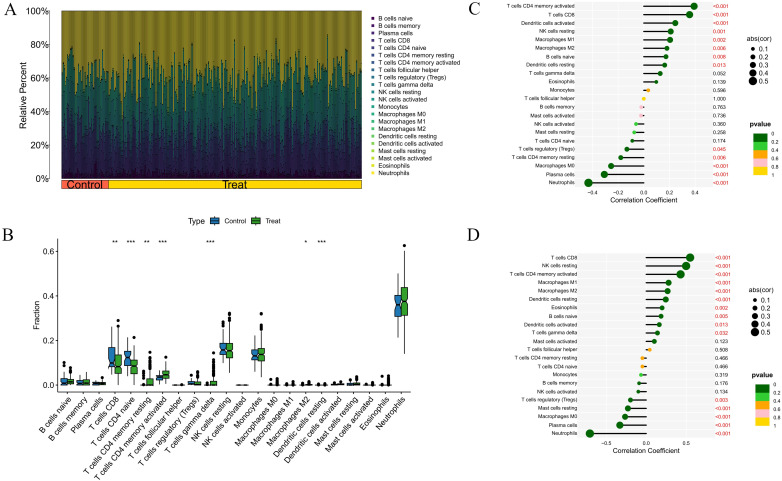
Immune landscape analysis in RA. **(A)** Histogram of proportion of immune cells. **(B)** Proportion of 22 kinds of immune cells in a boxplot diagram. **(C)** Correlation between HCP5 and immune cells content. **(D)** Correlation between RARRES3 and immune cells content. *p < 0.05; **p < 0.01;***p < 0.001.

**Fig 10:**
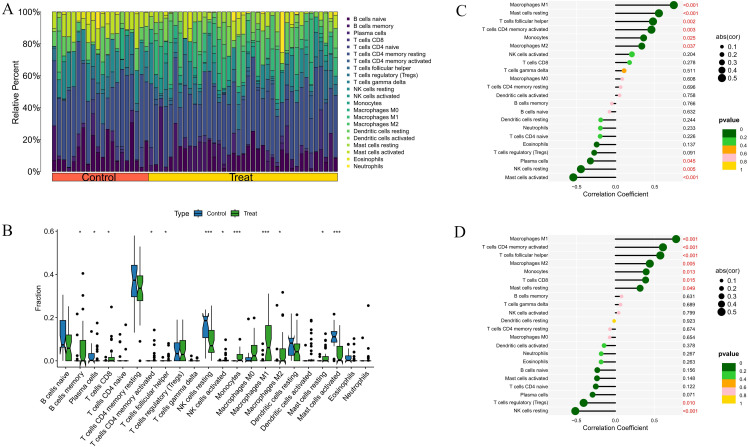
Immune landscape analysis in DM. **(A)** Histogram of proportion of immune cells. **(B)** Proportion of 22 kinds of immune cells in a boxplot diagram. **(C)** Correlation between HCP5 and immune cells content. **(D)** Correlation between RARRES3 and immune cells content. *p < 0.05; **p < 0.01;***p < 0.001.

We further explored the potential molecular mechanisms driving the progression of RA and DM in the immune microenvironment. We analyzed the percentage of 22 immune cell types in the RA and DM samples (Figs 9A, 10A). We observed higher proportions of CD4 + memory T cells and M2 macrophages in RA and DM disease groups, suggesting potential involvement in disease processes; however, functional assays and replication across cohorts are required to support causality or functional specificity (Figs 9B, 10B). Additionally, we analyzed the correlation between the core genes HCP5 and RARRES3 and immune cell content. In RA, both HCP5 and RARRES3 were positively correlated with M1 macrophages, M2 macrophages, CD8 + T cells, and resting NK cells, and negatively correlated with M0 macrophages, plasma cells, and neutrophils (Fig 9C, 9D). In DM, both HCP5 and RARRES3 were positively correlated with M1 macrophages and M2 macrophages, and negatively correlated with Tregs and resting NK cells (Fig 10C, 10D). These results were statistically significant (*P* < 0.05) and reveal the relationship between core genes and immune cells.

### Single-cell analysis results

**Fig 11:**
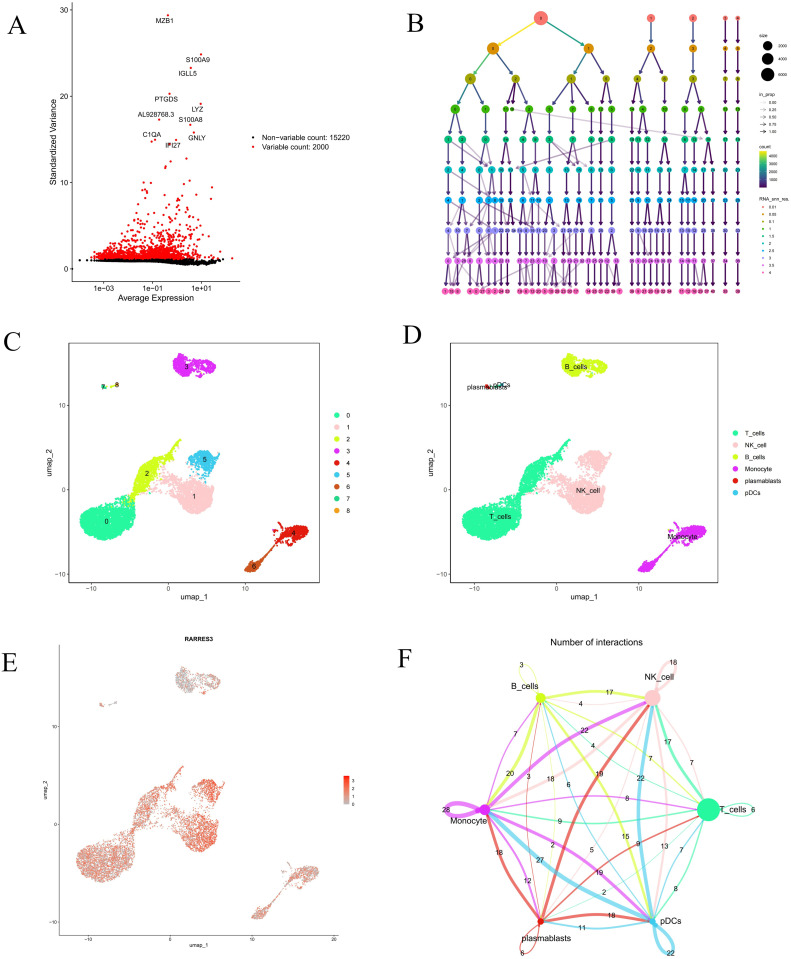
Analysis of single-cell RNA sequencing data from GSE159117. **(A)** Visualization of the top 10 highly variable genes in a volcano plot. **(B)** Select resolution (0.1). **(C, D)** U-map cell clustering distribution map for 6 cell subpopulations. **(E)** The UMAP plot displays the expression distribution of RARRES3 across various cell types. **(F)** Interaction number plot of synovial cells.

We performed preliminary quality control on the GSE159117 dataset by selecting 2,000 genes with significant differential expression and visualized the expression of the top 10 genes (Fig 11A). Next, we applied PCA for dimensionality reduction with a resolution of 0.1 (Fig 11B), dividing the core cell populations in the samples into nine independent clusters (Fig 11C). Based on this, we further annotated these clusters and identified six cell types: B cells, NK cells, T cells, monocytes, plasma cells, and pDCs, which were visually displayed using UMAP (Fig 11D). The analysis revealed that the expression of the RARRES3 gene was significantly higher in monocytes, T cells, and NK cells than in other cell types (Fig 11E). Additionally, we used the CellChat algorithm to construct a cross–cell-type signalling network model, focusing on the inferred ligand–receptor interaction patterns and their communication strength across cell clusters, and visualised the intercellular communication networks (Fig 11F).

## Discussion

In this study, we integrated transcriptomic analyses of RA and DM to uncover shared molecular mechanisms underlying these clinically distinct autoimmune disorders. We identified a convergent inflammatory signature characterized by up-regulation of pathways related to T helper 17 (Th17)/IL-17 signaling, Toll-like receptor activation, and chemokine-mediated leukocyte recruitment in both RA synovial tissue and DM muscle biopsies. Co-expression network analysis highlighted overlapping gene modules enriched in innate and adaptive immune response genes, aligning with an increased infiltration of pro-inflammatory immune cells in disease tissues. Immune deconvolution revealed that both RA and DM lesions harbor elevated levels of activated memory T lymphocytes (especially CD4^+^ memory T cells) and classically activated M1 macrophages. Notably, we pinpointed two hub genes, HCP5 and RARRES3, as central nodes in the shared RA–DM network. These genes were differentially expressed in both conditions and showed high connectivity in gene interaction networks, suggesting they may orchestrate critical inflammatory circuits common to RA and DM. Importantly, our major findings were validated in independent GEO cohorts, lending robustness to the observed cross-disease molecular commonalities. Taken together, the results indicate that despite different target tissues, RA and DM exhibit parallel immunopathogenic themes – including Th17/IL-17–driven inflammation, innate immune activation via TLR pathways, chemokine-driven leukocyte trafficking, and skewed immune cell composition – with HCP5 and RARRES3 emerging as potential shared regulatory hubs.

Our findings reinforce and extend current understanding of RA and DM pathogenesis by demonstrating a substantial mechanistic overlap rooted in chronic inflammation and dysregulated immune responses. IL-17/Th17 signaling emerged as a common thread, consistent with extensive evidence of this pathway’s pathogenic role in both diseases. In RA, IL-17 produced by Th17 cells acts on synovial fibroblasts and osteoclasts to perpetuate synovitis and joint destruction. Clinically, elevated IL-17A levels in synovium correlate with erosive disease, and IL-17 blockade has shown efficacy in related arthritides, underscoring IL-17’s contribution to RA’s chronic inflammation [39]. Likewise, DM – traditionally considered a humorally mediated myopathy – has now been shown to involve Th17 activity: patients with DM have increased muscle IL-17 and expanded Th17 cell populations, which associate with muscle fiber injury and disease severity. Li et al. demonstrated activation of the STAT3–IL-17 pathway in DM muscle, linking heightened Th17 responses to markers of muscle inflammation and dysfunction. This convergence on the IL-17 axis suggests that RA and DM, despite different end-organ targets (joint vs. muscle), share a T-cell–driven inflammatory cascade wherein IL-17 is a key effector cytokine driving local tissue damage in both settings [40].

Our data also implicate innate immune pathways, particularly TLR signaling, as a shared inflammatory driver in RA and DM. RA synovia are enriched with endogenous TLR ligands (e.g., heat-shock proteins, nucleic acids) and show heightened expression of TLRs on synovial macrophages and fibroblast-like cells. Engagement of TLRs in RA leads to persistent NF-κB and MAPK activation, fueling cytokine release (TNF, IL-6) and joint-destructive inflammation [41]. This aligns with the concept of RA as an autoimmune disease with a significant autoinflammatory component initiated by innate pattern recognition receptors. Similarly, in DM muscle tissues, damage-associated molecular patterns (DAMPs) released from injured fibers can activate TLRs and related innate sensors. Notably, HMGB1, an alarmin released in inflamed muscle, signals via TLR4 in DM, promoting immune cell activation and up-regulation of MHC class I on myofibers. Zong et al. reported that TLR4 is aberrantly expressed in DM muscle fibers and mediates HMGB1-induced muscle dysfunction and fatigue [42]. These observations parallel our finding of TLR pathway enrichment in both diseases and support a unifying model: ongoing innate immune activation (via TLR4 and others) sustains chronic inflammation in RA and DM even after the initial triggers (microbial or tissue injury) have passed. This may lead to a self-amplifying loop of inflammation, as endogenous TLR ligands generated by tissue damage perpetuate macrophage and dendritic cell activation in both joint and muscle environments [41]. By integrating our results with the literature, it becomes evident that RA and DM share a core “innate-adaptive” inflammatory axis: innate immune activation (TLRs, inflammasomes) sets the stage for and then synergizes with adaptive immune effectors (Th17 cells, autoantibodies), driving chronic tissue-specific injury.

Another prominent shared mechanism is chemokine-mediated leukocyte recruitment and retention in affected tissues. We found up-regulation of chemokine signaling pathways in both RA and DM, which is in line with the central role of chemokines in autoimmune inflammation. In RA, synovial cells and infiltrating leukocytes secrete an array of chemokines (CCL2, CCL5, CXCL8, CXCL10, CX3CL1, etc.) that mediate the ingress of monocytes, neutrophils, T cells, and B cells into the joint. This chemotactic network is a well-recognized driver of synovitis severity; for example, CXCL8 attracts neutrophils to the RA joint, whereas CXCL13 and CX3CL1 organize lymphoid aggregates and bring in T/B cells and patrolling monocytes [43]. In DM, recent transcriptomic and proteomic studies similarly point to chemokines as key immunopathogenic factors. Prior reports show that interferon-inducible CXC chemokines, notably CXCL10 (IP-10) and CXCL11, are highly expressed in DM and correlate with immune cell infiltration and disease activity. CXCL10/CXCL11 are potent chemoattractants for CXCR3^+^ T cells and NK cells, and their elevation in DM skin and muscle supports an interferon-γ–dominant, Th1-type milieu overlapping with the Th17 signature. Xu et al. identified CXCL10 and CXCL11 as top biomarkers reflecting the inflammatory burden in DM, particularly in patients with interstitial lung disease [44]. Both RA and DM thus exhibit a “chemokine storm” in affected tissues that sustains and compartmentalizes the inflammation – recruiting effector leukocytes into joints or muscles and contributing to tissue damage. This mechanistic overlap suggests that therapies targeting chemokine axes (e.g., CXCL10/CXCR3 blockade) might have utility across these diseases.

The immune cell composition findings from our study also align with known pathogenic immune cell subsets in RA and DM, strengthening the biological plausibility of shared pathways. We observed that CD4 + memory T cells are prominent in both RA synovium and DM muscle, together with increased CD8 + T cells and pro-inflammatory (M1) macrophages [45,46]. In RA, the synovium is indeed enriched for activated memory T cells (both CD4+ and CD8+) that orchestrate and propagate inflammation. These include Th1 and Th17 polarized cells that produce IFN-γ, IL-17, GM-CSF and other cytokines, contributing to macrophage activation and B-cell help. Sánchez-et al. noted that high levels of memory/effector T cells infiltrate RA joints, and both Th1 and Th17 subsets have been implicated in driving synovial inflammation [45]. Our results mirror this, and notably we saw a bias toward M1-polarized macrophages in RA synovia with a deficit of M2 macrophages. Macrophage polarization is critical: M1 macrophages produce IL-1β, TNF-α, and nitric oxide, mediating tissue damage, whereas M2 macrophages secrete IL-10 and aid repair. The predominance of M1 and relative reduction of M2 in RA is well documented and correlates with disease activity [46]. Strikingly, we found a similar inflammatory skew in DM: muscle lesions in DM show abundant infiltrating macrophages with an M1 phenotype and activated T cells [44,47]. Single-sample GSEA of DM tissues has revealed enrichment of multiple T cell subsets (CD4 + , CD8 + , γδ T cells, T_fh cells) and dendritic cells, alongside macrophages, indicating that DM muscles host a complex immune infiltrate analogous to RA synovium [47]. Recent DM studies highlight that macrophages in muscle can directly damage fibers and that an M1-rich environment (fostered by interferon-γ and TLR ligands) contributes to chronic myocyte injury [44]. The shared pattern of a Th1/Th17-T cell and M1-macrophage dominated infiltrate in both RA and DM underscores common immunological circuitry: an imbalance favoring pro-inflammatory effector cells over regulatory or pro-repair cells. This offers a unifying explanation for the persistent inflammation and tissue destruction seen in both diseases. Moreover, it suggests that immunomodulatory strategies aimed at restoring this balance – for instance, boosting regulatory T cells or promoting macrophage alternative activation – could have cross-disease therapeutic value.

Crucially, our integrative analysis highlighted HCP5 (HLA complex P5) and RARRES3 (retinoic acid receptor responder 3) as central hub genes linking RA and DM, providing mechanistic context for shared disease processes. HCP5 is a long non-coding RNA (lncRNA) located in the MHC class I region and has emerged as an immune-regulatory lncRNA relevant to autoimmunity [48]. Consistent with our findings, HCP5 expression is elevated in RA synovial tissues and correlates with immune-cell infiltration. Xiao et al. identified HCP5 as a key up-regulated lncRNA in RA and reported positive associations with infiltrating CD8 + T cells, γδ T cells, and M1 macrophages [46], suggesting induction in inflammatory milieus and a potential role in sustaining the inflammatory infiltrate. Mechanistically, HCP5 can act as a competing endogenous RNA (ceRNA), sponging specific microRNAs and thereby modulating immune-related gene expression. Kulski noted that HCP5 functions as a regulatory hub by interacting with microRNAs and transcriptional regulators within immune-response pathways [48]. Supporting this regulatory capacity, in a neonatal sepsis model, HCP5 overexpression sponged miR-138-5p, up-regulated SIRT1, and attenuated macrophage production of TNF-α and IL-8 [49], illustrating how HCP5 can influence macrophage activation and cytokine release via lncRNA–miRNA interactions. By analogy, in chronic RA and DM inflammation, HCP5 may modulate key inflammatory checkpoints by buffering immune-regulatory miRNAs, thereby reinforcing cytokine production and immune-cell survival. HCP5 has also been implicated in other autoimmune contexts [48], further supporting its immunomodulatory role. Together, our identification of HCP5 as a shared hub gene suggests it may link MHC-associated genetic risk to downstream inflammatory gene regulation in both RA and DM, potentially through ceRNA-mediated effects on T-cell and macrophage phenotypes.

RARRES3 (also known as TIG3 or PLAAT4) was another hub gene bridging RA and DM, and its known functions provide insight into potential shared counter-regulatory mechanisms. RARRES3 is induced by retinoic acid and p53 and has been described as a class II tumour suppressor that limits proliferation and promotes differentiation [50]. It encodes a protein with phospholipase A1/2 and acyltransferase activity, enabling modification of lipid moieties on signalling proteins [51], which can reshape inflammatory signalling. Notably, RARRES3 antagonises the Wnt/β-catenin and PI3K/Akt pathways, both implicated in immune activation and tissue inflammation. Lee et al. showed that RARRES3 promotes deacylation and intracellular retention of Wnt proteins, suppressing Wnt/β-catenin signalling and downstream targets [51]. RARRES3 can also inhibit H-Ras activation and dampen PI3K/Akt and MAPK cascades associated with pro-inflammatory phenotypes. In RA, Wnt/β-catenin hyperactivation in synovial fibroblasts supports pannus formation and joint destruction, while PI3K/Akt signalling promotes immune-cell survival and cytokine production. Reduced RARRES3 expression could therefore remove a brake on these pathways, facilitating synoviocyte proliferation and inflammatory-cell persistence [52,53], whereas up-regulation may reflect feedback restraint in specific contexts. RARRES3 has also been identified as an interferon-stimulated gene with antimicrobial functions downstream of IRF1 and type I/II interferons [50]. Given the prominent type I interferon signatures in DM, particularly in clinically amyopathic DM and anti-MDA5-positive disease, RARRES3 may be induced as part of this response [54] and could help limit muscle injury by constraining inflammatory signalling within the muscle microenvironment.

In summary, integrating our findings with existing literature suggests that RA and DM, despite distinct target organs, share a core pathogenic framework characterised by sustained Th17/IL-17 activity, innate immune activation, and chemokine-driven inflammatory-cell infiltration. Identifying HCP5 and RARRES3 as shared hub genes provides insight into the regulatory architecture underpinning this inflammation: HCP5 may modulate immune responses post-transcriptionally via lncRNA–miRNA interactions, whereas RARRES3 may attenuate pro-inflammatory signalling (e.g., Wnt and PI3K/Akt) within retinoic acid and interferon regulatory circuits. Together, these hubs highlight multi-layered regulation of chronic inflammation in RA and DM and nominate potential cross-disease therapeutic targets, including IL-17-, TLR4-, CXCL10-, HCP5-, and Wnt/β-catenin–related pathways.

A key strength of this study is the systems-biology approach we employed, leveraging multiple public gene expression datasets and complementary analytical methods to dissect the RA–DM connection. We first identified differentially expressed genes separately in RA and DM, and then integrated these with disease-associated co-expression modules using WGCNA, so that the final set of overlapping candidate genes captured both strong differential expression and network-level association with disease. Functional enrichment of these overlapping genes, together with immune cell deconvolution, consistently pointed to shared pro-inflammatory and immune-regulatory pathways, providing convergent support for common pathogenic mechanisms between RA and DM. This multi-pronged strategy reduces the bias inherent in any single method and allows internal cross-validation of results. Another strength is the use of independent validation cohorts for both diseases: we discovered overlapping signatures in primary datasets and then confirmed key findings, including the hub status of HCP5 and RARRES3, in separate patient cohorts or datasets. This strengthens confidence that the shared mechanisms are not dataset-specific artefacts but reflect robust disease biology.

We acknowledge several important limitations. First, differences in tissue context (synovium vs. muscle) and experimental platforms between the RA and DM datasets pose a challenge. While we interpreted common pathways, the baseline transcriptomic profiles of joint and muscle differ substantially. Some observed differences or lack of overlap could be due to tissue-specific gene expression rather than true disease disparities. We attempted to mitigate this by focusing on immune and inflammatory genes (which are more likely to be shared across tissues), but this remains a limitation – for instance, muscle fibers in DM can produce unique factors (like muscle-specific enzymes or structural proteins) that have no counterpart in RA synovium. Second, the sample sizes in the analyzed datasets, though moderate, are not large (e.g., 10 RA vs 10 controls in GSE55235; 36 DM vs 20 controls in GSE143323) and may limit statistical power. The heterogeneity of patient populations (varying disease durations, activity, and treatments) could also have influenced the results. RA and DM are clinically heterogeneous – for example, RA ranges from seropositive erosive disease to seronegative mild disease, and DM has distinct subsets (classic vs. amyopathic, different autoantibody profiles). Our analysis did not stratify these subgroups; thus, the shared signature we report may preferentially reflect certain subsets (such as aggressive RA or classic DM) and might not be uniformly present in all patients. Third, our approach was observational and based on bulk tissue transcriptomics. This means we cannot ascribe the gene expression changes to specific cell types with certainty. We used computational deconvolution (CIBERSORT, ssGSEA) to estimate immune cell infiltration, but these methods have inherent limitations and potential errors. Without single-cell resolution data, there is a risk of confounding – for example, increased HCP5 expression in RA synovium could mean more HCP5 per cell or simply more immune cells that happen to express HCP5. Additionally, network analyses (WGCNA, hub gene identification) can highlight associations but not causality. We cannot conclude from correlation alone that HCP5 or RARRES3 cause the observed inflammatory features; experimental validation (e.g., gene knockdown or overexpression in disease models) is needed to establish functional roles. Another limitation is that our pathway enrichment analyses relied on curated databases (KEGG, GO), which may not capture context-specific pathway crosstalk. Some pathways (e.g., “chemokine signaling”) are broad and could oversimplify distinct chemokine interactions in RA vs. DM. Moreover, certain relevant pathways might not have been identified due to multiple-testing corrections or because they are disease-specific (for instance, we might have under-detected the Type I interferon signature in DM, a well-known aspect of DM immunopathology, because it’s less prominent in RA). It’s worth noting that we did observe interferon-inducible chemokines and IRF1-related genes (like RARRES3) in DM, but a dedicated analysis of interferon-stimulated genes might have revealed even more overlap or differences. Finally, while we identified HCP5 and RARRES3 as intriguing hub genes, these findings rely on bioinformatic criteria (connectivity, differential expression) and previously published associations. We labeled some references in our discussion as repeated (where we re-used evidence from prior work to support our interpretation), which could introduce bias if those references were part of our initial knowledge base. Experimental limitations of the original datasets (e.g., batch effects, microarray probe issues for lncRNAs like HCP5) could also affect our results. In summary, our study provides hypotheses and associative links but is limited in the ability to prove mechanistic involvement; targeted experiments in cellular or animal models of RA and DM will be required to validate the roles of the shared pathways and hub genes we identified.

Future work should adopt a cross-disease, causality-focused agenda that combines single-cell and spatial multi-omics with CRISPR or pharmacological perturbation to test how HCP5 and RARRES3 regulate Th17, TLR and chemokine programmes in defined cell types, and validate findings in prospective cohorts. Clinically, priority should be given to biomarker-guided repurposing trials that stratify patients by immune phenotype rather than diagnosis, for example evaluating IL-17 or JAK inhibitors and TLR4/MyD88 pathway blockers in DM, while developing compact panels such as CXCL10, IL-17A and HCP5/RARRES3 to monitor activity and response. At the policy level, regulators and funders should encourage pathway-driven trial designs that enrol across diseases on the basis of molecular signatures, and support interdisciplinary clinics with shared registries and real-world data pipelines to accelerate translation and reproducibility.

## Conclusion

Through integrative analysis of multiple GEO datasets, we delineated convergent inflammatory programmes shared by RA and DM, including up-regulated Th17/IL-17, Toll-like receptor and chemokine signalling, alongside an immune-cell landscape enriched for activated memory T cells and M1 macrophages relative to M2. Network and machine-learning analyses nominated HCP5 and RARRES3 as candidate shared hubs, supported in independent cohorts and showing moderate diagnostic performance, thereby generating testable hypotheses for cross-disease biomarkers and mechanisms.

## Supporting information

S1 File **(A)** The determination of soft thresholding power in RA dataset. **(B)** The determination of soft thresholding power in DM dataset.
(PDF)

S2 File Validation of the expression level of JUNB, NRGN. (A) The violin plots of JUNB in RA dataset. (B) The violin plots of JUNB in DM dataset. (C) The violin plots of NRGN in RA dataset. (D) The violin plots of NRGN in DM dataset. (PDF)

## References

[1] Lundberg IE, Fujimoto M, Vencovsky J, Aggarwal R, Holmqvist M, Christopher-Stine L, et al. Idiopathic inflammatory myopathies. Nat Rev Dis Primers. 2021;7(1):86. doi: 10.1038/s41572-021-00321-x 34857798

[2] Smolen JS, Aletaha D, McInnes IB. Rheumatoid arthritis. Lancet. 2016;388(10055):2023–38. doi: 10.1016/S0140-6736(16)30173-8 27156434

[3] Baker MC, Liu Y, Lu R, Lin J, Melehani J, Robinson WH. Incidence of Interstitial Lung Disease in Patients With Rheumatoid Arthritis Treated With Biologic and Targeted Synthetic Disease-Modifying Antirheumatic Drugs. JAMA Netw Open. 2023;6(3):e233640. doi: 10.1001/jamanetworkopen.2023.3640 36939701 PMC10028485

[4] Baechler EC, Bilgic H, Reed AM. Type I interferon pathway in adult and juvenile dermatomyositis. Arthritis Res Ther. 2011;13(6):249. doi: 10.1186/ar3531 22192711 PMC3334651

[5] Lin CMA, Isaacs JD, Cooles FAH. Role of IFN-α in Rheumatoid Arthritis. Curr Rheumatol Rep. 2024;26(2):37–52. doi: 10.1007/s11926-023-01125-6 38051494 PMC10787895

[6] Cooles FAH, Isaacs JD. The interferon gene signature as a clinically relevant biomarker in autoimmune rheumatic disease. Lancet Rheumatol. 2022;4(1):e61–72. doi: 10.1016/S2665-9913(21)00254-X 38288732

[7] Lahoria R, Selcen D, Engel AG. Microvascular alterations and the role of complement in dermatomyositis. Brain. 2016;139(Pt 7):1891–903. doi: 10.1093/brain/aww122 27190020

[8] Yell PC, Burns DK, Dittmar EG, White CL 3rd, Cai C. Diffuse microvascular C5b-9 deposition is a common feature in muscle and nerve biopsies from diabetic patients. Acta Neuropathol Commun. 2018;6(1):11. doi: 10.1186/s40478-018-0512-6 29458425 PMC5819078

[9] Padyukov L. Genetics of rheumatoid arthritis. Semin Immunopathol. 2022;44(1):47–62. doi: 10.1007/s00281-022-00912-0 35088123 PMC8837504

[10] Leclair V, Galindo-Feria AS, Rothwell S, Kryštůfková O, Zargar SS, Mann H, et al. Distinct HLA associations with autoantibody-defined subgroups in idiopathic inflammatory myopathies. EBioMedicine. 2023;96:104804. doi: 10.1016/j.ebiom.2023.104804 37769433 PMC10550566

[11] Jani M, Massey J, Wedderburn LR, Vencovský J, Danko K, Lundberg IE, et al. Genotyping of immune-related genetic variants identifies TYK2 as a novel associated locus for idiopathic inflammatory myopathies. Ann Rheum Dis. 2014;73(9):1750–2. doi: 10.1136/annrheumdis-2014-205440 24812289 PMC4471138

[12] Morand E, Merola JF, Tanaka Y, Gladman D, Fleischmann R. TYK2: an emerging therapeutic target in rheumatic disease. Nat Rev Rheumatol. 2024;20(4):232–40. doi: 10.1038/s41584-024-01093-w 38467779

[13] Mizoguchi F, Slowikowski K, Wei K, Marshall JL, Rao DA, Chang SK, et al. Functionally distinct disease-associated fibroblast subsets in rheumatoid arthritis. Nat Commun. 2018;9(1):789. doi: 10.1038/s41467-018-02892-y 29476097 PMC5824882

[14] Culemann S, Grüneboom A, Nicolás-Ávila JÁ, Weidner D, Lämmle KF, Rothe T, et al. Locally renewing resident synovial macrophages provide a protective barrier for the joint. Nature. 2019;572(7771):670–5. doi: 10.1038/s41586-019-1471-1 31391580 PMC6805223

[15] Sehgal S, Patel A, Chatterjee S, Fernandez AP, Farver C, Yadav R, et al. Idiopathic inflammatory myopathies related lung disease in adults. Lancet Respir Med. 2025;13(3):272–88. doi: 10.1016/S2213-2600(24)00267-4 39622261 PMC13138733

[16] Narváez J. Moving forward in Rheumatoid Arthritis-Associated Interstitial Lung Disease Screening. J Clin Med. 2024;13(18):5385. doi: 10.3390/jcm13185385 39336873 PMC11432920

[17] Chatterjee S. MDA5 dermatomyositis: Unveiling a potentially life-threatening disease. Cleve Clin J Med. 2025;92(10):627–37. doi: 10.3949/ccjm.92a.25018 41033848

[18] Kurtzman DJB, Vleugels RA. Anti-melanoma differentiation-associated gene 5 (MDA5) dermatomyositis: A concise review with an emphasis on distinctive clinical features. J Am Acad Dermatol. 2018;78(4):776–85. doi: 10.1016/j.jaad.2017.12.010 29229575

[19] Nombel A, Fabien N, Coutant F. Dermatomyositis with anti-MDA5 antibodies: bioclinical features, pathogenesis and emerging therapies. Front Immunol. 2021;12:773352. 34745149 10.3389/fimmu.2021.773352 PMC8564476

[20] Ramiro S, Nikiphorou E, Sepriano A, Ortolan A, Webers C, Baraliakos X, et al. ASAS-EULAR recommendations for the management of axial spondyloarthritis: 2022 update. Ann Rheum Dis. 2023;82(1):19–34. doi: 10.1136/ard-2022-223296 36270658

[21] Chen Z, Wang X, Ye S. Tofacitinib in Amyopathic Dermatomyositis-Associated Interstitial Lung Disease. N Engl J Med. 2019;381(3):291–3. doi: 10.1056/NEJMc1900045 31314977

[22] Zhao Q, Zhu Z, Fu Q, Shih Y, Wu D, Chen L, et al. Baricitinib for the treatment of cutaneous dermatomyositis: A prospective, open-label study. J Am Acad Dermatol. 2022;87(6):1374–6. doi: 10.1016/j.jaad.2022.08.025 35998841

[23] Leek JT, Johnson WE, Parker HS, Jaffe AE, Storey JD. The sva package for removing batch effects and other unwanted variation in high-throughput experiments. Bioinformatics. 2012;28(6):882–3. doi: 10.1093/bioinformatics/bts034 22257669 PMC3307112

[24] Conesa A, Madrigal P, Tarazona S, Gomez-Cabrero D, Cervera A, McPherson A, et al. A survey of best practices for RNA-seq data analysis. Genome Biol. 2016;17:13. doi: 10.1186/s13059-016-0881-8 26813401 PMC4728800

[25] Ritchie ME, Phipson B, Wu D, Hu Y, Law CW, Shi W, et al. limma powers differential expression analyses for RNA-sequencing and microarray studies. Nucleic Acids Res. 2015;43(7):e47. doi: 10.1093/nar/gkv007 25605792 PMC4402510

[26] Chen Y, Lun ATL, Smyth GK. From reads to genes to pathways: differential expression analysis of RNA-Seq experiments using Rsubread and the edgeR quasi-likelihood pipeline. F1000Res. 2016;5:1438. doi: 10.12688/f1000research.8987.2 27508061 PMC4934518

[27] Bardou P, Mariette J, Escudié F, Djemiel C, Klopp C. jvenn: an interactive Venn diagram viewer. BMC Bioinformatics. 2014;15(1):293. doi: 10.1186/1471-2105-15-293 25176396 PMC4261873

[28] Wan Q, Tang J, Han Y, Wang D. Co-expression modules construction by WGCNA and identify potential prognostic markers of uveal melanoma. Exp Eye Res. 2018;166:13–20. doi: 10.1016/j.exer.2017.10.007 29031853

[29] Yu G, Wang L-G, Han Y, He Q-Y. clusterProfiler: an R package for comparing biological themes among gene clusters. OMICS. 2012;16(5):284–7. doi: 10.1089/omi.2011.0118 22455463 PMC3339379

[30] Reimand J, Isserlin R, Voisin V, Kucera M, Tannus-Lopes C, Rostamianfar A, et al. Pathway enrichment analysis and visualization of omics data using g:Profiler, GSEA, Cytoscape and EnrichmentMap. Nat Protoc. 2019;14(2):482–517. doi: 10.1038/s41596-018-0103-9 30664679 PMC6607905

[31] Hu J, Szymczak S. A review on longitudinal data analysis with random forest. Brief Bioinform. 2023;24(2):bbad002. doi: 10.1093/bib/bbad002 36653905 PMC10025446

[32] Grimes DA. The nomogram epidemic: resurgence of a medical relic. Ann Intern Med. 2008;149(4):273–5. doi: 10.7326/0003-4819-149-4-200808190-00010 18711159

[33] Kattan MW, Marasco J. What is a real nomogram? Semin Oncol. 2010;37(1):23–6. doi: 10.1053/j.seminoncol.2009.12.003 20172360

[34] Bifarin OO. Interpretable machine learning with tree-based shapley additive explanations: Application to metabolomics datasets for binary classification. PLoS One. 2023;18(5):e0284315. doi: 10.1371/journal.pone.0284315 37141218 PMC10159207

[35] Sender R, Weiss Y, Navon Y, Milo I, Azulay N, Keren L, et al. The total mass, number, and distribution of immune cells in the human body. Proc Natl Acad Sci U S A. 2023;120(44):e2308511120. doi: 10.1073/pnas.2308511120 37871201 PMC10623016

[36] Mangiola S, Doyle MA, Papenfuss AT. Interfacing Seurat with the R tidy universe. Bioinformatics. 2021;37(22):4100–7. doi: 10.1093/bioinformatics/btab404 34028547 PMC9502154

[37] Huang Q, Liu Y, Du Y, Garmire LX. Evaluation of Cell Type Annotation R Packages on Single-cell RNA-seq Data. Genomics Proteomics Bioinformatics. 2021;19(2):267–81. doi: 10.1016/j.gpb.2020.07.004 33359678 PMC8602772

[38] Jin S, Guerrero-Juarez CF, Zhang L, Chang I, Ramos R, Kuan C-H, et al. Inference and analysis of cell-cell communication using CellChat. Nat Commun. 2021;12(1):1088. doi: 10.1038/s41467-021-21246-9 33597522 PMC7889871

[39] Robert M, Miossec P. IL-17 in Rheumatoid Arthritis and Precision Medicine: From Synovitis Expression to Circulating Bioactive Levels. Front Med (Lausanne). 2019;5:364. doi: 10.3389/fmed.2018.00364 30693283 PMC6339915

[40] Li D, Jia W, Zhou L, Hao Y, Wang K, Yang B, et al. Increased expression of the p-STAT3/IL-17 signaling pathway in patients with dermatomyositis. Mod Rheumatol. 2023;34(1):129–36. doi: 10.1093/mr/roac147 36478263

[41] Huang Q-Q, Pope RM. The role of toll-like receptors in rheumatoid arthritis. Curr Rheumatol Rep. 2009;11(5):357–64. doi: 10.1007/s11926-009-0051-z 19772831 PMC2913446

[42] Zong M, Bruton JD, Grundtman C, Yang H, Li JH, Alexanderson H, et al. TLR4 as receptor for HMGB1 induced muscle dysfunction in myositis. Ann Rheum Dis. 2013;72(8):1390–9. doi: 10.1136/annrheumdis-2012-202207 23148306

[43] Bodolay E, Koch AE, Kim J, Szegedi G, Szekanecz Z. Angiogenesis and chemokines in rheumatoid arthritis and other systemic inflammatory rheumatic diseases. J Cell Mol Med. 2002;6(3):357–76. doi: 10.1111/j.1582-4934.2002.tb00514.x 12417052 PMC6740222

[44] Xu X, Qiu T, Sun K, Han X, Huang J, Wang X, et al. Integrated analysis of dermatomyositis reveals heterogeneous immune infiltration and interstitial lung disease-associated endotype. Arthritis Res Ther. 2025;27(1):26. doi: 10.1186/s13075-025-03494-y 39923079 PMC11806601

[45] Mellado M, Martínez-Muñoz L, Cascio G, Lucas P, Pablos JL, Rodríguez-Frade JM. T cell migration in rheumatoid arthritis. Front Immunol. 2015;6:384. 26284069 10.3389/fimmu.2015.00384 PMC4515597

[46] Xiao J, Cai X, Huang X, Guo F, Chen X, Hong Y, et al. The expression of long non-coding RNA human leukocyte antigen complex P5(lncRNA HCP5) in synovial tissue of patients with rheumatoid arthritis is up-regulated and correlated with immune cell infiltration. Xi Bao Yu Fen Zi Mian Yi Xue Za Zhi. 2023;39(5):445–50. 37248839

[47] Wang S, Tang Y, Chen X, Song S, Chen X, Zhou Q, et al. Mitochondrial-related hub genes in dermatomyositis: muscle and skin datasets-based identification and in vivo validation. Front Genet. 2024;15:1325035. doi: 10.3389/fgene.2024.1325035 38389573 PMC10882082

[48] Kulski JK. Long Noncoding RNA HCP5, a Hybrid HLA Class I Endogenous Retroviral Gene: Structure, Expression, and Disease Associations. Cells. 2019;8(5):480. doi: 10.3390/cells8050480 31137555 PMC6562477

[49] Hu X, Hu A, Luo Y, Yuan S, Yang L. LncRNA HCP5 acts as a potential diagnostic biomarker and attenuates the inflammatory response in neonatal sepsis by targeting miR-138-5p/SIRT1. Cent Eur J Immunol. 2024;49(3):216–26. doi: 10.5114/ceji.2024.143462 39720269 PMC11664802

[50] Zhao J-Y, Yuan X-K, Luo R-Z, Wang L-X, Gu W, Yamane D, et al. Phospholipase A and acyltransferase 4/retinoic acid receptor responder 3 at the intersection of tumor suppression and pathogen restriction. Front Immunol. 2023;14:1107239. doi: 10.3389/fimmu.2023.1107239 37063830 PMC10102619

[51] Hsu T-H, Chang T-C. RARRES3 regulates signal transduction through post-translational protein modifications. Mol Cell Oncol. 2015;2(4):e999512. doi: 10.1080/23723556.2014.999512 27308522 PMC4905368

[52] Riitano G, Spinelli F, Manganelli V, Caissutti D, Capozzi A, Garufi C, et al. Wnt signaling as a translational target in rheumatoid and psoriatic arthritis. J Transl Med. 2025;23(1):158. doi: 10.1186/s12967-025-06174-2 39905450 PMC11796213

[53] Voorzanger-Rousselot N, Ben-Tabassi NC, Garnero P. Opposite relationships between circulating Dkk-1 and cartilage breakdown in patients with rheumatoid arthritis and knee osteoarthritis. Ann Rheum Dis. 2009;68(9):1513–4. doi: 10.1136/ard.2008.102350 19674991

[54] Cassius C, Amode R, Delord M, Battistella M, Poirot J, How-Kit A, et al. MDA5+ Dermatomyositis Is Associated with Stronger Skin Type I Interferon Transcriptomic Signature with Upregulation of IFN-κ Transcript. J Invest Dermatol. 2020;140(6):1276-1279.e7. doi: 10.1016/j.jid.2019.10.020 31955963

