# Supplementary material for: Identification of common genes and biomarkers between Dermatomyositis and rheumatoid arthritis through integrated bioinformatics
Source: PLoS One. 2026 Feb 4;21(2):e0340617. doi: 10.1371/journal.pone.0340617 (PMC12872010; doi:10.1371/journal.pone.0340617)
Supplement: S1 File — (A) The determination of soft thresholding power in RA dataset. (B) The determination of soft thresholding power in DM dataset. (PDF) [file pone.0340617.s001.pdf]

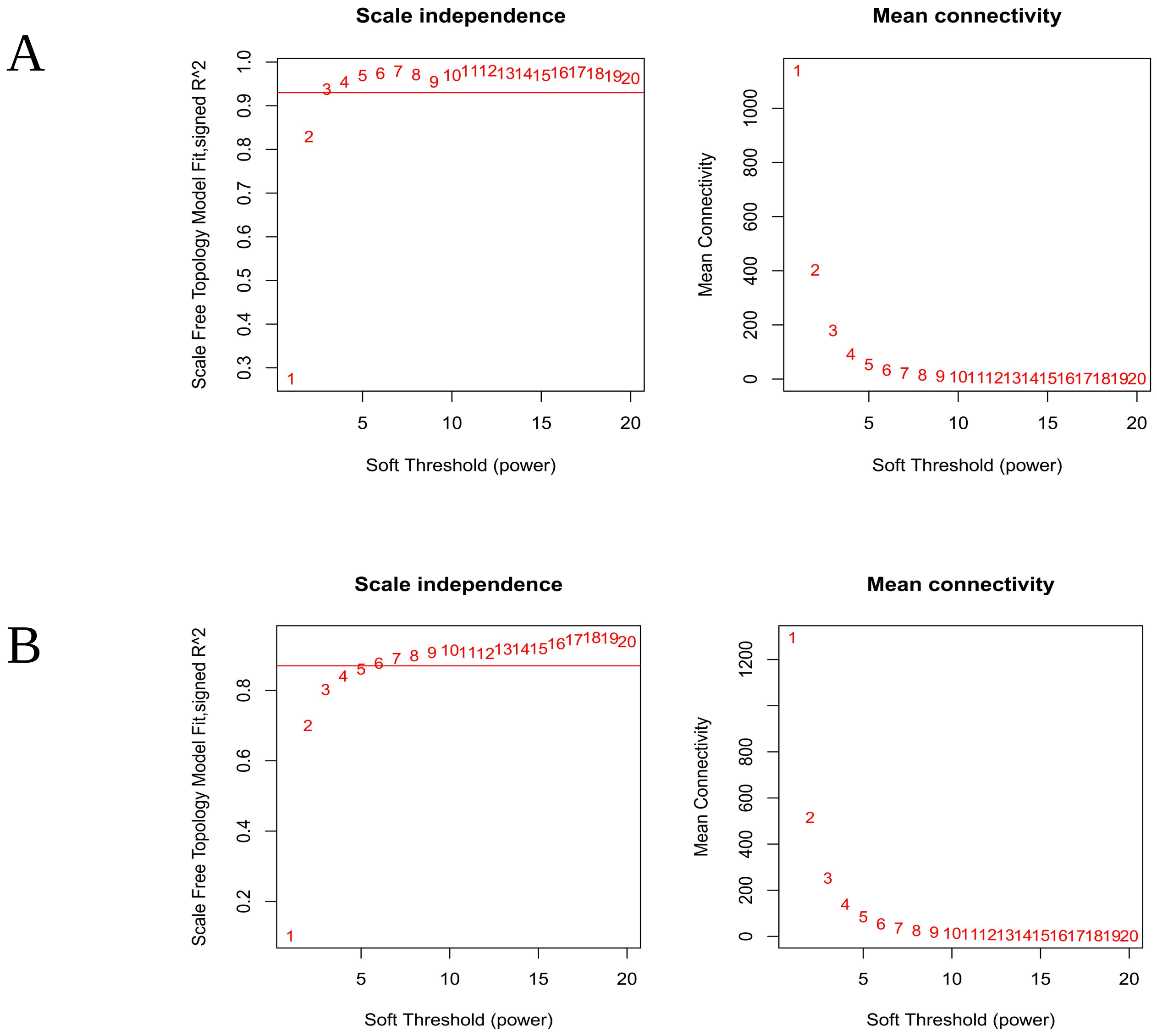

Supplementary File 1: (A) The determination of soft thresholding power in RA dataset. (B) The determination of soft thresholding power in DM dataset.
