# Supplementary material for: Identification of common genes and biomarkers between Dermatomyositis and rheumatoid arthritis through integrated bioinformatics
Source: PLoS One. 2026 Feb 4;21(2):e0340617. doi: 10.1371/journal.pone.0340617 (PMC12872010; doi:10.1371/journal.pone.0340617)
Supplement: S2 File — (PDF) [file pone.0340617.s002.pdf]

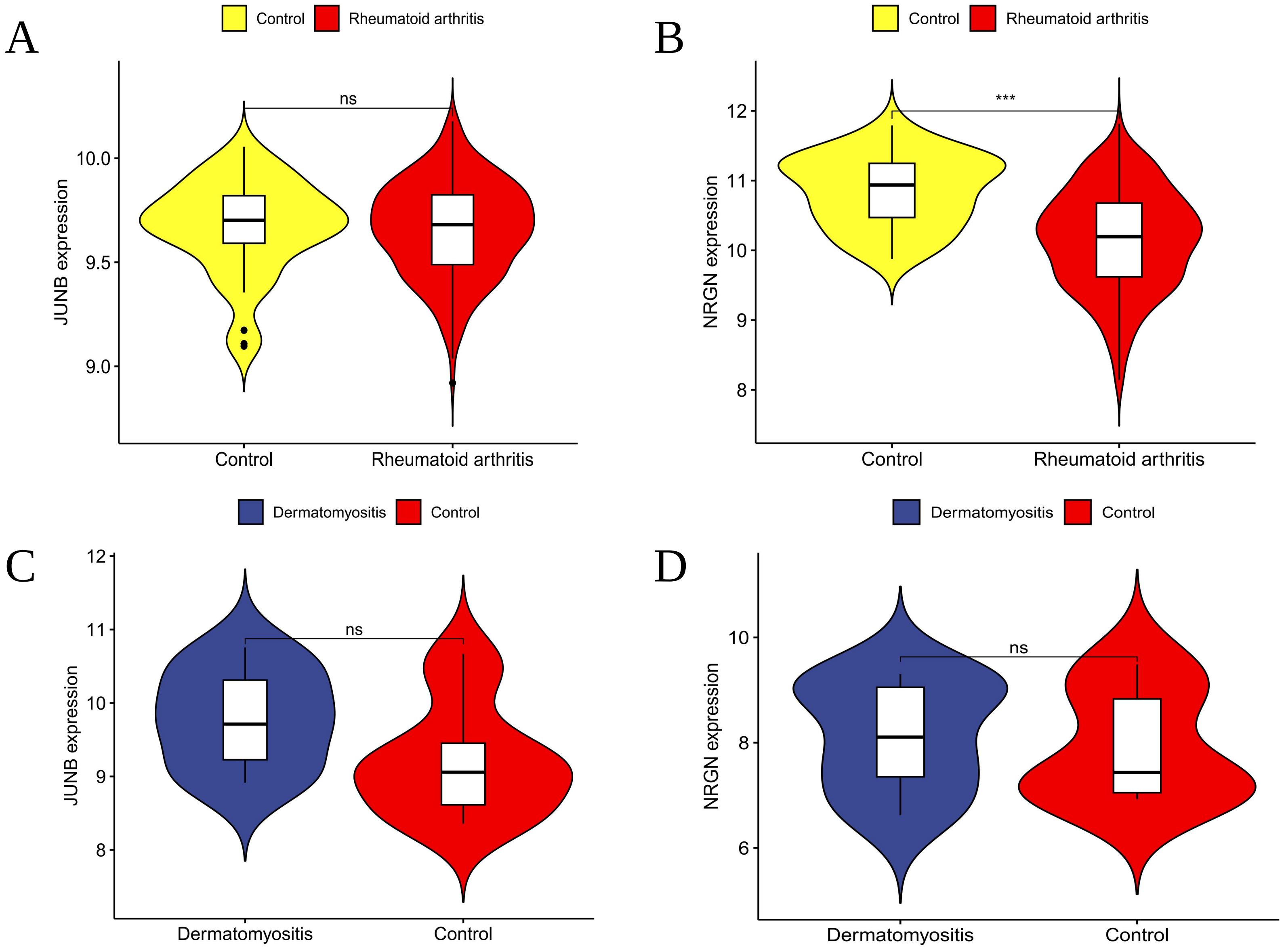

Supplementary File 2: Validation of the expression level of JUNB, NRGN. (A) The violin plots of JUNB in RA dataset. (B) The violin plots of JUNB in DM dataset. (C) The violin plots of NRGN in RA dataset. (D) The violin plots of NRGN in DM dataset.
